# Supplementary material for: Validation of automated paper screening for esophagectomy systematic review using large language models
Source: PeerJ Comput Sci. 2025 Apr 30;11:e2822. doi: 10.7717/peerj-cs.2822 (PMC12190591; doi:10.7717/peerj-cs.2822)
Supplement: Supplemental Information 1 — (Appendix A) literature search conducted with the help of trained librarian and (Appendix B) inclusion/exclusion approach for both iterations. [file peerj-cs-11-2822-s001.docx]

**Supplementary Appendix**

Embase Classic+Embase <1947 to 2023 February 21>

Ovid MEDLINE(R) ALL <1946 to February 21, 2023>

EBM Reviews - Cochrane Central Register of Controlled Trials <January 2023>

1 Esophagectomy/ 14381

2 (esophagectom* or oesophagectom*).tw,kf. 36911

3 1 or 2 41153

4 postoperative complications/ or anastomotic leak/ 565021

5 ((postoper* or post operat* or post surg*) adj3 complication*).tw,kf. 311304

6 (anastomo* adj2 leak*).tw,kf. 34330

7 (multiple adj2 dilatation*).tw,kf. 685

8 Dilatation/ 36804

9 Constriction, Pathologic/ 42358

10 (stricture* or stenosis).tw,kf. 552037

11 Intubation, Gastrointestinal/ 15246

12 ((nasogastric or gastrointest*) adj2 (tube* or intubat*)).tw,kf. 20748

13 Gastric Emptying/ 35082

14 delay* gastric empt*.tw,kf. 12545

15 Pneumonia, Aspiration/ 18951

16 (aspiration adj2 (pneumonia* or syndrome*)).tw,kf. 19457

17 Mendelson* Syndrome*.tw,kf. 513

18 j tube*.tw,kf. 660

19 Jejunostom* tube*.tw,kf. 1258

20 feeding tube*.tw,kf. 12955

21 Feeding jejunostom*.tw,kf. 1412

22 Pylorus/ and Drainage/ 262

23 (pylor* adj2 drainag*).tw,kf. 199

24 pyloromyoplast*.tw,kf. 14

25 or/4-24 1452981

26 3 and 25 14848

27 risk assessment/ or risk factors/ 2699464

28 risk*.ti. 1518400

29 (risk adj2 (factor* or assessment or stratification* or score*)).tw,kf. 2296371

30 (((patient* or perioper* or preoper*) adj2 risk*) or predict*).tw,kf. 5161571

31 or/27-30 8642211

32 26 and 31 3323

33 exp animals/ not humans/ 17545705

34 32 not 33 2880

35 34 use medall 1511

36 exp esophagectomy/ 15345

37 (esophagectom* or oesophagectom*).tw. 36142

38 36 or 37 40629

39 postoperative complication/ 822711

40 anastomosis leakage/ 25606

41 ((postoper* or post operat* or post surg*) adj3 complication*).tw. 296428

42 (anastomo* adj2 leak*).tw. 33802

43 dilatation/ 36804

44 (multiple adj2 dilatation*).tw. 685

45 stricture*.mp. or stenosis.tw. 540907

46 nasogastric tube/ 15399

47 ((nasogastric or gastrointest*) adj2 (tube* or intubat*)).tw. 19740

48 stomach paresis/ 10389

49 delay* gastric empt*.tw. 12444

50 aspiration pneumonia/ 24732

51 (aspiration adj2 (pneumonia* or syndrome*)).tw. 18955

52 Mendelson* Syndrome*.tw. 502

53 j tube*.tw. 650

54 Jejunostom* tube*.tw. 1245

55 feeding tube*.tw. 12746

56 exp feeding tube/ 28214

57 Feeding jejunostom*.tw. 1401

58 (pylor* adj3 drainag*).tw. 261

59 pyloromyotomy/ or pyloroplasty/ 4696

60 pyloromyoplast*.tw. 14

61 or/39-60 1620407

62 38 and 61 15549

63 risk assessment/ or risk factor/ 3023890

64 risk*.ti. 1518400

65 (risk adj2 (factor* or assessment or stratification* or score*)).tw. 2229048

66 (((patient* or perioper* or preoper*) adj2 risk*) or predict*).tw. 5143875

67 or/63-66 8688074

68 62 and 67 3487

69 (exp animal/ or nonhuman/) not exp human/ 12984987

70 conference abstract.pt. 4690982

71 68 not (69 or 70) 2996

72 71 use emczd 1474

73 Esophagectomy/ 14381

74 (esophagectom* or oesophagectom*).tw,kw. 36835

75 73 or 74 41087

76 postoperative complications/ or anastomotic leak/ 565021

77 ((postoper* or post operat* or post surg*) adj3 complication*).tw,kw. 304675

78 (anastomo* adj2 leak*).tw,kw. 34081

79 (multiple adj2 dilatation*).tw,kw. 685

80 Dilatation/ 36804

81 Constriction, Pathologic/ 42358

82 (stricture* or stenosis).tw,kw. 535356

83 Intubation, Gastrointestinal/ 15246

84 ((nasogastric or gastrointest*) adj2 (tube* or intubat*)).tw,kw. 20130

85 Gastric Emptying/ 35082

86 delay* gastric empt*.tw,kw. 12543

87 Pneumonia, Aspiration/ 18951

88 (aspiration adj2 (pneumonia* or syndrome*)).tw,kw. 19307

89 Mendelson* Syndrome*.tw,kw. 513

90 j tube*.tw,kw. 656

91 Jejunostom* tube*.tw,kw. 1271

92 feeding tube*.tw,kw. 12947

93 Feeding jejunostom*.tw,kw. 1412

94 Pylorus/ and Drainage/ 262

95 (pylor* adj2 drainag*).tw,kw. 204

96 pyloromyoplast*.tw,kw. 14

97 or/76-96 1438093

98 75 and 97 14751

99 risk assessment/ or risk factors/ 2699464

100 risk*.ti. 1518400

101 (risk adj2 (factor* or assessment or stratification* or score*)).tw,kw. 2250307

102 (((patient* or perioper* or preoper*) adj2 risk*) or predict*).tw,kw. 5161625

103 or/99-102 8626099

104 98 and 103 3300

105 conference proceeding.pt. 214901

106 104 not 105 3293

107 106 use cctr 63

108 35 or 72 or 107 3048

109 remove duplicates from 108 1969

**Appendix 1:** Detailed Search Strategy from OVID and Medline. This appendix contains the detailed literature search strategy and terms used. This appendix is provided for transparency, reproducibility, and verification of our systematic literature search. For a simplified description of the search approach, please refer to the Methods section in the main manuscript.

Run #1: Peri-operative risk factor

*Inclusion Criteria:*

1. Studies focusing on patients who underwent esophagectomy for any indication (e.g., cancer, benign disease).
2. Studies reporting on perioperative risk factors associated with anastomotic complications (e.g., anastomotic leaks, strictures) following esophagectomy.
3. Randomized controlled trials (RCTs), cohort studies, case-control studies, or prospective observational studies.
4. Articles published in English language.
5. Studies with adult participants (age ≥18 years).

*Exclusion Criteria:*

1. Studies not reporting specific perioperative risk factors for anastomotic complications.
2. Case reports, reviews, letters, editorials, and conference abstracts.
3. Studies focusing solely on pediatric populations.
4. Studies with insufficient data or incomplete reporting.
5. Studies not relevant to the investigation of anastomotic complications after esophagectomy.
6. Studies published before a specified date (if applicable).
7. Non-human studies or studies conducted on cadavers.
8. Duplicate publications or multiple reports from the same study.

Run #2: Pre-operative risk factors

*Inclusion Criteria:*

1. Studies focusing on patients who underwent esophagectomy for any indication (e.g., cancer, benign disease).
2. Studies focusing on pre-operative risk factors associated with anastomotic complications (e.g., leaks, strictures) following esophagectomy.
3. Randomized controlled trials (RCTs), cohort studies, case-control studies, or prospective observational studies.
4. Articles published in the English language.
5. Studies with adult participants (age ≥18 years).

*Exclusion Criteria:*

1. Studies not reporting specific pre-operative risk factors for anastomotic complications.
2. Case reports, reviews, letters, editorials, and conference abstracts.
3. Studies focusing solely on pediatric populations.
4. Studies with insufficient data or incomplete reporting.
5. Studies not relevant to the investigation of pre-operative risk factors for anastomotic complications after esophagectomy.
6. Studies published before a specified date (if applicable).
7. Non-human studies or studies conducted on cadavers.
8. Duplicate publications or multiple reports from the same study.

**Appendix 2:** Inclusion and Exclusion criteria for perioperative and preoperative title and abstract screening
